# Design and evaluation of a health education prompt list using network analysis for patients after PCI

**DOI:** 10.3389/fpubh.2025.1740551

**Published:** 2026-01-12

**Authors:** Fang Zhen, Songmei Cao, Jingjing Wang, Qin Yuan, Xiaoyan Zhao, Liu Jun

**Affiliations:** Department of Nursing, The Affiliated Hospital of Jiangsu University, Zhenjiang, China

**Keywords:** health education, network analysis, PCI, percutaneous coronary intervention, prompt list

## Abstract

**Objective:**

To develop a checklist of health education prompts for patients after coronary heart disease interventional procedures, and to employ network analysis to identify core issues, thereby providing a structured tool for patients to actively acquire health information.

**Methods:**

From January to June 2024, items were generated through literature review, patient interviews (*n* = 8), and two rounds of expert correspondence (*n* = 12). A questionnaire survey was then administered to 200 patients. Principal component analysis and network analysis were combined to identify problem clusters and core issues.

**Results:**

The final checklist comprised 8 first-level items and 20 s-level items. Network analysis revealed a modular structure of the problems, with “Coronary Heart Disease Risk Factors” and “Postoperative Complications of Coronary Heart Disease” identified as the core clusters.

**Conclusion:**

The developed checklist of health education prompts for patients after coronary heart disease interventional procedures is clinically applicable. The use of network analysis elucidated the modular structure and interrelationships within post-procedural health education for coronary heart disease. It can assist nursing staff in providing targeted health education, enhancing patients’ postoperative self-management capabilities, and offers direct guidance for nursing practice.

## 1 Introduction

Coronary heart disease (CHD) represents a major global public health challenge, with its morbidity and mortality rates ranking among the highest of all cardiovascular diseases (1). In China, the number of CHD patients has reached 11.39 million, and since 2012, both the incidence and mortality rates have shown a continuous upward trend (2). Percutaneous coronary intervention (PCI) has become a primary treatment for CHD due to its proven efficacy, minimal invasiveness, and short recovery times (3, 4). However, PCI does not reverse the pathological progression of atherosclerosis. Risk factors such as hypertension and diabetes often persist after the procedure, and some patients struggle with poor medication adherence and difficulties in managing their lifestyles (5–7). As a result, cases of recurrent angina pectoris or even rehospitalization within a short period after surgery are not uncommon. For these patients, standardizing medication use and lifestyle through systematic health education is particularly important. However, existing health education models primarily rely on in-hospital group sessions, which fail to address long-term management after discharge. Moreover, their uniform approach, broad content, and lack of personalization often lead to suboptimal learning outcomes for patients (8). Additionally, while encouraging patients to ask questions is a key way to involve them in treatment decision-making, in reality, insufficient access to information and low levels of actual participation in decision-making significantly hinder both their willingness and ability to pose questions (9).

To address this communication challenge, the Question Prompt List (QPL) was introduced as an effective communication tool. Pre-established questions and topics were utilized during clinical consultations to guide and facilitate in-depth discussions between healthcare providers and patients concerning treatment and care (10). Patient engagement and self-management capabilities throughout the treatment process were enhanced by the QPL (11), enabling them to gain a more comprehensive understanding of their condition and treatment options. Consequently, information was actively exchanged, and deeper involvement in treatment decision-making was achieved.

Structured informational frameworks were demonstrated to be provided to patients through QPLs, with positive impacts on communication quality being observed across various patient populations (12, 13). Zietlow et al. (14) utilized a question prompt list to conduct structured health education for older preoperative patients, ensuring that older adults have a realistic understanding of surgery, risks, and recovery. Tsai et al. (15) demonstrated that clinicians can incorporate the QPL into routine health education for breast cancer patients. By providing a pre-structured list of potentially relevant questions, the QPL reduces the psychological and cognitive barriers for breast cancer patients to initiate questions. It grants patients the “legitimacy” and concrete ideas for asking questions, encouraging them to shift from passive recipients of information to active communication participants, thereby enhancing their self-efficacy and sense of agency in medical decision-making. Munoz et al. (16) suggested that the QPL may address the challenges faced by Latinx parents with limited English proficiency (LEP) during the transition home. Question prompt lists can serve as suggested questions for families to use in health education, improving family-centered communication. Standardized discharge instructions may not meet all patients’ needs. The QPL promotes personalized health education, laying a more solid foundation for long-term self-management after discharge.

However, standardized manuals or question prompt lists primarily developed based on cross-sectional surveys present a simple linear arrangement and fail to capture the complex interdependencies between psychosocial factors and health behaviors. Recent studies (17) have highlighted the limitations of questionnaire-based methods in identifying unmet needs, as they often overlook the dynamic nature of patient concerns. To address this gap, this study innovatively employs network analysis to visualize the intricate relationships among patient concerns. This method allows for an in-depth examination of the intrinsic connections and structure among complex symptoms or problems from a “network” perspective, and identifies core elements, thereby further enhancing the precision and efficiency of health education (18–20). With the growing demand for health education among patients with coronary heart disease, network analysis has been demonstrated to effectively improve the effectiveness and quality of health management for chronic conditions (21, 22). Therefore, this study applies principal component analysis and network analysis to conduct an in-depth examination of the constructed Health Education Prompt List for post-PCI patients. Through the symptom network paradigm, it aims to scientifically identify problem clusters and core issues in health education for this population, thereby providing a basis for optimizing intervention strategies.

## 2 Materials and methods

### 2.1 Participants

#### 2.1.1 Interview subjects

This study employed purposive sampling and adhered to the principle of data saturation. Patients who had undergone percutaneous coronary intervention for coronary heart disease and were discharged from a tertiary hospital between March and April 2024 were selected as pre-interview subjects. The inclusion criteria were: (I) age ≥18 years; (II) meeting the diagnostic criteria for coronary heart disease; (III) having successfully undergone PCI; (IV) being conscious with adequate comprehension and verbal expression abilities; and (V) being informed of and voluntarily agreeing to participate in the study. The exclusion criteria included: (I) presence of other severe organic diseases; and (II) presence of communication disorders, cognitive impairments, or language barriers.

#### 2.1.2 Delphi experts

The expert panel members were recruited through purposive sampling combined with snowball sampling. Based on established selection criteria (more than 10 years of experience in the diagnosis, treatment, or nursing of coronary heart disease; holding an intermediate or above professional technical title; possessing a bachelor’s degree or higher), 12 experts were invited to participate in the Delphi study. The working years of the expert team ranged from 11 to 33 years, and the composition included 4 clinical physicians and 8 nursing experts. The distribution of professional titles was as follows: 1 full senior title, 8 associate senior titles, and 3 intermediate titles. The educational background consisted of 3 doctors, 3 masters, and 6 bachelors.

#### 2.1.3 Survey participants

A total of 200 patients with coronary heart disease who underwent PCI during their hospitalization in the cardiology department of a tertiary hospital between January and June 2024 were selected as study subjects using convenience sampling. The inclusion criteria were as follows: (I) age ≥18 years, with informed consent and voluntary participation; (II) meeting the diagnostic criteria for coronary heart disease; (III) successful PCI treatment; and (IV) clear consciousness with adequate comprehension and verbal expression abilities. The exclusion criteria were as follows: (1) presence of other severe organic diseases; and (2) presence of communication disorders, cognitive impairments, or language barriers. The age of the participants ranged from 31 to 88 years, with a mean age of 31.30 ± 3.80 years. The educational background distribution was as follows: associate degree or higher, 22 cases (11.0%); high school education, 35 cases (17.5%); junior high school education, 91 cases (45.5%); and primary school education or below, 52 cases (26.0%). The medical history profile included: hypertension, 139 cases (69.5%); diabetes, 49 cases (24.5%); smoking history, 88 cases (44.0%); and alcohol consumption history, 58 cases (29.0%).

### 2.2 Study design

This study adopted a mixed-methods design, integrating qualitative and quantitative research in stages to develop and preliminarily evaluate a health education prompt list. The main phases included: collecting preliminary health issues through literature analysis and individual in-depth interviews; using the Delphi expert consultation method to screen and reach consensus on the issues; developing a survey tool based on the consensus results and conducting a cross-sectional survey of post-PCI patients to obtain data on the occurrence of issues; and finally, employing network analysis to identify core clusters of health problems.

### 2.3 Data collection procedures

#### 2.3.1 Literature analysis

Health issues in patients after PCI were collected through literature analysis. A systematic search was conducted using keywords including “coronary disease*,” “myocardial ischemia,” “angina pectoris,” “myocardial infarction,” “percutaneous coronary intervention,” and “percutaneous coronary revascularization.” Data sources encompassed guideline databases and organizations such as Best Practice, the National Guideline Clearinghouse, the Guidelines International Network, the Scottish Intercollegiate Guidelines Network, and the UK National Institute for Health and Care Excellence. Additional resources included the Registered Nurses’ Association of Ontario, the World Health Organization, the American Heart Association, the American College of Cardiology, the European Society of Cardiology, the British Association for Cardiovascular Prevention and Rehabilitation, and comprehensive databases like PubMed, Web of Science, and EMBASE. The search covered all records from database inception to December 2023, focusing on guidelines, systematic reviews, or meta-analyses related to post-PCI care. Two researchers independently reviewed the full texts of the included literature and extracted health issues relevant to post-PCI patients into a standardized Excel spreadsheet.

#### 2.3.2 In-depth individual interviews

This study was approved by the Scientific Research Ethics Committee of the Affiliated Hospital of Jiangsu University (KY2025H0926–11) and received consent from the research management department of the Cardiovascular Medicine Department at the Affiliated Hospital of Jiangsu University. Questionnaires were collected by the principal investigator and two postgraduate students from the research team. All researchers received standardized training prior to the survey, and the principal investigator explained all items of the questionnaire to avoid ambiguity in understanding among the researchers.

Using purposive sampling and guided by the principle of information saturation, patients who had undergone coronary intervention for coronary heart disease and were discharged from a tertiary hospital between March and April 2024 were selected as interviewees. After obtaining informed consent, one-on-one in-depth interviews were conducted with the participants in a quiet meeting room. A semi-structured interview outline served as the framework for flexible questioning, and the interview process remained objective and neutral, without leading the respondents. Each interview lasted 10 to 30 min, with an average duration of 16 min. Audio recordings were made with the participants’ consent. The recordings were transcribed verbatim by a trained postgraduate student, and all transcripts were cross-checked against the original recordings to ensure data accuracy.

After confirming that no new critical information emerged, two additional patients were recruited using the same criteria, confirming that data saturation had been reached. In total, eight patients were included. Their demographic and clinical characteristics are as follows: age range 49–74 years (mean 63.25 ± 11.88 years); 7 males and 1 female; 2 cases of chronic coronary heart disease, 5 cases of acute myocardial infarction, and 1 case of unstable angina; 5 cases with hypertension, 3 cases with diabetes; 3 smokers, 1 alcohol consumer; education levels: 1 university graduate, 3 high school graduates, 2 middle school graduates, and 2 primary school graduates; no religious beliefs. The interview data were analyzed using thematic analysis.

#### 2.3.3 Delphi consultation

The expert consultation questionnaires were distributed to the experts through a questionnaire application, comprising four sections: (1) Questionnaire Instructions: introducing the research background and objectives, and explaining the method for completing the questionnaire; (2) QPL Content Evaluation Form: employing a 5-point Likert scale to assess the importance of each item in the prompt list, along with providing suggestions for additions, deletions, or modifications; (3) Expert Basic Information Form: including eight items such as name, age, and professional title; and (4) Expert Familiarity and Judgment Basis: self-assessment by experts regarding their familiarity with the health needs of post-PCI patients and the basis for their judgments. After each round of consultation, expert feedback was collected and consolidated, and the questionnaire items were supplemented and revised accordingly based on the consultation results. After two rounds of consultation, expert opinions reached consensus, and a pre-survey questionnaire was finalized. If ≥75% of the panel members rated an item as “strongly agree” or “agree,” it was directly included in the final health education list (23). Items for which experts provided important modification suggestions were discussed and revised by the research team before proceeding to the next round of evaluation. Items considered inapplicable or redundant by more than 30% of the experts in the qualitative feedback were removed. Each round of expert consultation concluded either after all panel members had responded or after a two-week period. At the end of each Delphi round, experts received a feedback report containing the statistical results of that round (mean scores, standard deviations, and CVs for each item), their own ratings from the previous round, and anonymized summaries of modification suggestions for reference in the next round of judgment.

#### 2.3.4 Questionnaire survey

Based on the health issues identified through literature review and expert consultation, a Health Problem Survey for post-PCI discharged patients was developed. Each item in the survey required a “Yes” or “No” response, asking patients whether they had encountered these nursing problems after discharge. Subjects meeting the inclusion and exclusion criteria were selected from the cardiology department of a tertiary hospital. Data collection was conducted through outpatient follow-up surveys and telephone interviews, where the survey purpose was explained and informed consent was obtained. A total of 220 questionnaires were distributed, with 200 valid responses collected, yielding an effective response rate of 90.91%.

### 2.4 Statistical analysis

In this study, SPSS 26.0 software was used for descriptive statistical analysis. Qualitative data were presented as frequencies and percentages (%). Quantitative data that followed a normal distribution were expressed as mean ± standard deviation, while those that did not were presented as median and interquartile range. The engagement level of the experts was indicated by the questionnaire response rate and the rate at which they provided suggestions. The authority coefficient (Cr) of the experts was calculated using the coefficient of judgment basis (Ca) and the coefficient of familiarity (Cs), with Cr = (Ca + Cs)/2. The degree of consensus among experts was represented by the mean importance score and the full score ratio of each item. The degree of coordination of expert opinions was measured using the coefficient of variation and Kendall’s coefficient of concordance. The significance level was set at *α* = 0.05. Principal component analysis with varimax rotation was employed to extract factors with eigenvalues >1 and factor loadings >0.4. This was used to reduce the dimensionality of the initial health education items and explore their underlying latent structure, thereby forming clusters of postoperative health issues. To describe the relationships among these health issues and issue clusters, network analysis was performed using R. The qgraph package was utilized to construct the network diagram, while centrality measures (including strength centrality, closeness centrality, and betweenness centrality) were applied to precisely identify the most central postoperative health issues in the network. The stability of the network was assessed using the Bootnet package (correlation stability coefficient should be at least 0.25, preferably greater than 0.5) (24).

## 3 Results

### 3.1 Results of literature analysis

A total of 59 publications were included, comprising 10 clinical guidelines and 49 systematic reviews/Meta-analyses. Fifty issues related to health education after coronary heart disease interventional surgery were initially extracted, which were subsequently refined through consolidation and refinement into 22 core questions. These questions span eight key themes: disease-related knowledge, surgical-related knowledge, medication management, dietary management, exercise management, symptom management, emotional management, and disease self-management. Among these themes, “disease self-management” emerged as the most frequently addressed area in the literature, while “emotional management” was the least frequently discussed topic.

### 3.2 Results of in-depth individual interviews

Through interviews with 8 discharged patients who had undergone percutaneous coronary intervention, 42 post-PCI health issues were identified. The data were organized and analyzed using the qualitative research analysis software Nvivo 14.0. The extraction and summarization of textual themes strictly followed the six-step thematic analysis method proposed by Braun et al. (25) Subsequently, based on the conceptual content of these issues, the results from both literature analysis and interviews underwent deduplication, consolidation, and summarization. Based on the principle of including only issues mentioned by patients and supported by the literature, 20 health problems were screened to form the Health Problem List for post-PCI patients.

### 3.3 Data integration

In the first phase, the research team conducted a comparative review. We mapped the themes identified in the literature with those derived from the interviews and performed semantic similarity comparisons on the items. If a theme existed in both sources (e.g., “diet management”), we verified whether its definitions were consistent. Discrepancies were resolved by revisiting the original textual evidence (literature citations vs. interview transcripts). The second phase applied a consolidation strategy based on the principle that “patient voices are supported by evidence.” Items were categorized into three types: Convergent Items, which were identified in both sources (e.g., “healthy eating”). These items were retained as high-priority. Exclusive Items - Literature Only, which were present only in the literature but absent from patient interviews (e.g., highly technical surgical details). To ensure practicality, these items were excluded. Exclusive Items - Interview Only, which were reported by patients but lacked sufficient literature support (e.g., specific emotional distress). If these items were considered critical to patient safety, they were retained but flagged for further validation. This iterative process ultimately yielded a final list of 20 items, balancing clinical evidence with patient needs.

### 3.4 Results of Delphi consultation

#### 3.4.1 Expert response rate

In the first round, 12 questionnaires were distributed, all of which were effectively returned, resulting in a 100% effective response rate. In the second round, 12 questionnaires were distributed, all of which were also effectively returned, achieving a 100% effective response rate. The response rates for both consultation rounds exceeded 70%, indicating a high level of positive engagement among the experts.

#### 3.4.2 Expert authority level

The expert judgment coefficients for the two consultation rounds were 0.941 and 0.95 respectively, while the expert familiarity coefficients were 0.88 and 0.87, respectively. The coefficient of variation in the first round was 0.05 to 0.26. The coefficient of variation in the second round was 0.05 to 0.18. The expert authority coefficients, calculated based on the judgment and familiarity coefficients, were 0.91 and 0.90, respectively. An authority coefficient (Cr) greater than 0.70 indicates a high level of expert authority, thus confirming the high expert authority level in this consultation round.

#### 3.4.3 Expert coordination degree

The Kendall’s coefficients for the two consultation rounds were 0.228 and 0.249, respectively (χ2 = 64.871, 53.805, *p* < 0.01 for both), indicating good consistency among expert opinions and reliable results. The coordination coefficient showed improvement in the second round compared to the first, demonstrating that the revised items in the second round, based on feedback from the initial consultation, received greater consensus from the experts. As expert opinions converged satisfactorily, the consultation process was concluded.

### 3.5 Questionnaire survey

#### 3.5.1 Results of principal component analysis

The questionnaire demonstrated a Cronbach’s alpha coefficient of 0.963. The KMO measure was 0.902, and Bartlett’s test of sphericity was significant (*p* < 0.01), indicating the suitability of the data for principal component analysis (PCA). Through PCA, three main components were extracted, including Basic Disease Knowledge, Lifestyle Management, and Symptom Management. The rotated component matrix results indicated that items 1, 2, 3, 4, 5, 6, 7, 8, and 18 loaded on the first dimension, representing the Basic Disease Knowledge dimension; items 9, 10, 11, 12, 13, 14, 15, 19, and 20 loaded on the Lifestyle Management dimension; while items 16 and 17 loaded on the Symptom Management dimension.

#### 3.5.2 Results of network Analysi

**Figure 1:**
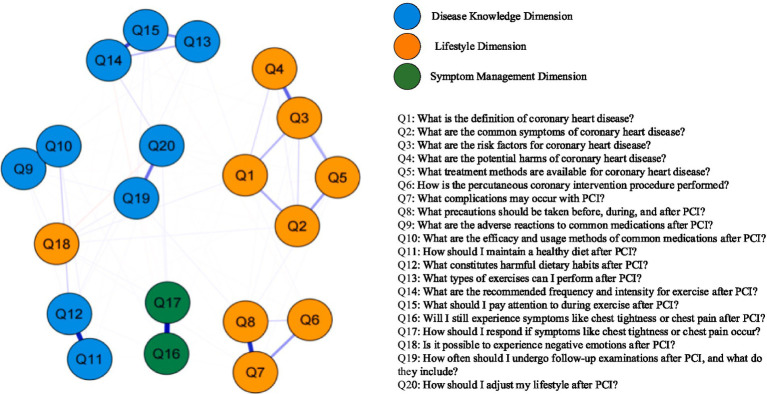
Network of health issues in patients after coronary intervention.

In the network structure model, thicker edges with darker colors between symptoms within a cluster indicate stronger correlations among them. As shown in Figure 1, the strongest correlations are observed between Q16 and Q17, Q7 and Q8, and Q11 and Q12. In the network diagram, blue connecting lines represent positive correlations between two items, while pink lines indicate negative correlations. It can be observed that most post-PCI health issues demonstrate positive correlations with each other.

**Figure 2:**
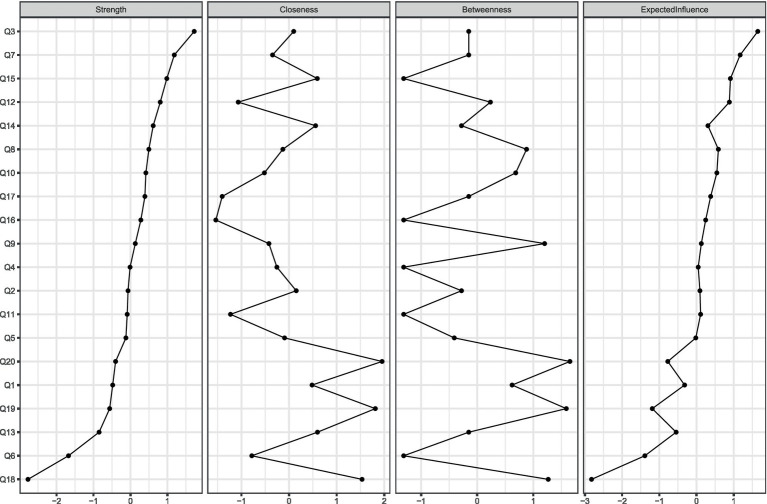
Centrality index of the network of health issues in patients after coronary intervention.

As shown in Figure 2, “Risk factors for coronary heart disease” and “Potential complications of PCI” demonstrated the highest strength centrality within the node network, indicating these are core concerns in the post-PCI symptom clusters. This suggests that most patients lack comprehensive knowledge regarding disease-related information. Similarly, “Whether negative emotions will occur after PCI,” “Follow-up schedule and content after PCI,” and “Lifestyle adjustments after PCI” achieved the highest values in both closeness and betweenness centrality. This indicates that these three issues are positioned closest to other health education concerns in the network and serve as the most critical bridges within the entire post-PCI health education problem cluster. Further evaluation of the network’s stability and accuracy was conducted. The correlation stability coefficients (CS-coefficients) for closeness and betweenness were 0.005 and 0.67, respectively. The CS-coefficient for closeness was below 0.25, indicating poor stability of this measure. In contrast, the CS-coefficient for strength was 0.75, demonstrating good stability. Therefore, based on the correlation stability coefficient for strength, the network exhibits satisfactory stability, indicating high accuracy in both edge weights and centrality measures.

**Figure 3:**
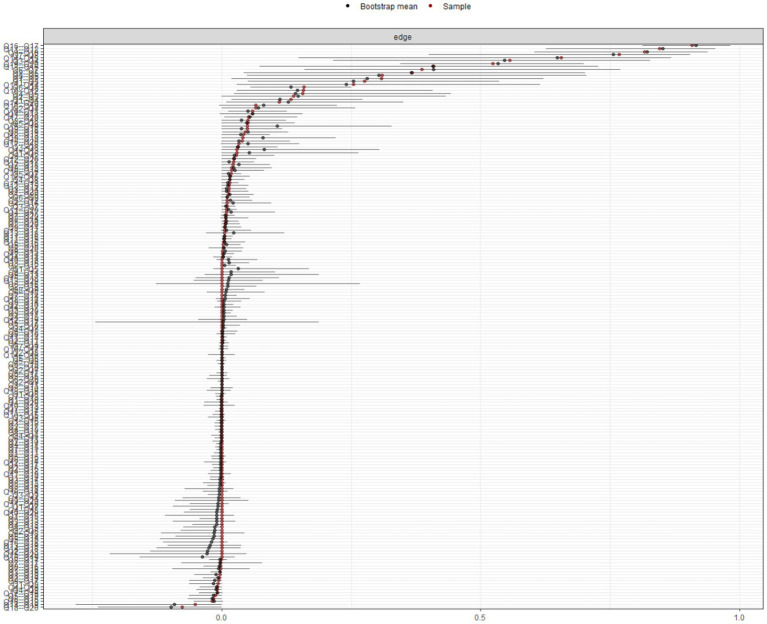
Accuracy of edge weight in the network of health issues in patients after coronary intervention.

**Figure 4:**
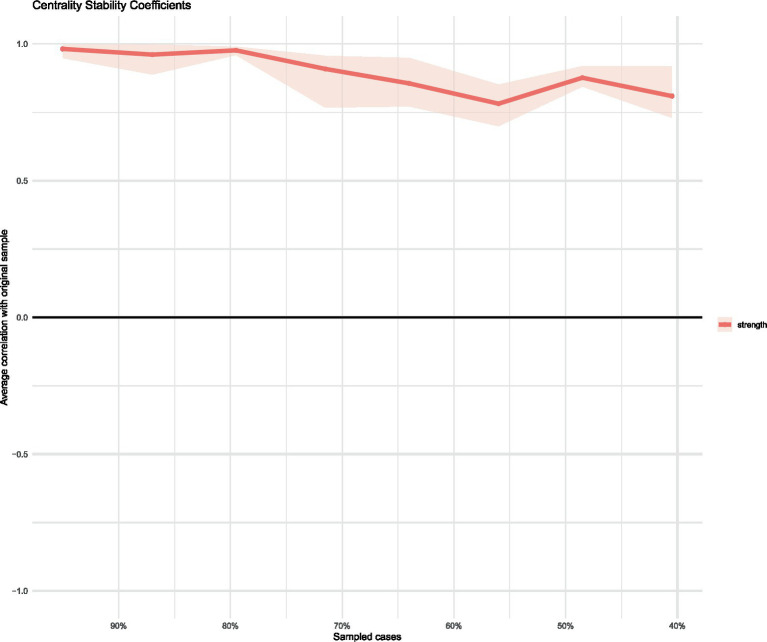
Stability in the network of health issues in patients after coronary intervention.

The stability analysis presented in Figures 3, 4 demonstrates that the network model is stable. Specifically, the edge weight stability analysis indicates that the connection strengths are reliable. Among the centrality stability measures, strength centrality shows the highest stability.

## 4 Discussion

### 4.1 Significance of constructing a health education prompt list for post-PCI patients

QPL holds significant clinical practical value and theoretical innovative significance. As a communication tool between healthcare providers and patients, it enables medical staff to initiate dialogues through structured questions and provide targeted responses based on patients’ inquiries, thereby enhancing patients’ understanding of disease-related knowledge. The Health Education Prompt List for post-PCI patients systematically integrates medical evidence and clinical experience, providing healthcare professionals with a structured health education framework. Previous studies have predominantly developed educational content based on the supply-side of healthcare (such as guidelines and expert consensus) (26) or relied solely on questionnaires to assess patient needs (17). This study innovatively applies principal component analysis to scientifically categorize question items, forming a structured system encompassing three dimensions: foundational disease knowledge, lifestyle adjustment, and symptom management. This classification not only comprehensively covers patients’ postoperative knowledge needs but also aligns closely with clinical practice, serving as a significant supplement to traditional methods of health education content development. This structure clearly maps the complete cycle of PCI postoperative rehabilitation—from “cognition” to “behavior” and then to “monitoring”—and aligns well with the core domains of chronic disease self-management. Compared to the fragmented knowledge points provided in some previous studies, this structure helps patients establish a systematic cognitive framework before discharge. This standardized system not only helps reduce variability in health education outcomes caused by differences in individual healthcare provider expertise but also promotes the process of standardized education, thereby enhancing the stability of overall nursing quality.

### 4.2 Significance of conducting network analysis on the question prompt list

This study identified risk factors and surgical complications as the most critical concerns for patients after PCI, forming the central structure in the health problem network of coronary heart disease patients. Multiple studies (27, 28) indicate that patients’ insufficient understanding of the root causes of coronary heart disease and the risks associated with interventional treatment is a key barrier to poor adherence in secondary prevention. Insufficient understanding of risk factors represents one of the key reasons for poor lifestyle adherence and failure in secondary prevention of coronary heart disease. Only when patients comprehend the negative impacts of factors such as hypertension, diabetes, and smoking on post-PCI recovery can they enhance their motivation for self-management and maintain long-term lifestyle adjustments. Simultaneously, with improved knowledge of surgical complications, patients can better assess surgical risks, cooperate with pre-, intra-, and post-operative care (as evidenced by the strong correlation between Q7 and Q8), while avoiding negative emotions caused by minor post-operative discomfort. Therefore, these two aspects should serve as key entry points in health education to enhance both the efficiency and quality of educational interventions.

“Will I experience negative emotions after PCI?” (Q18), “How often should I undergo follow-up examinations after PCI and what do they entail?” (Q19), and “How should I adjust my lifestyle after PCI?” (Q20) – these three nodes achieved the highest values in both closeness and betweenness centrality. High closeness indicates that the sum of distances from this node to all other nodes in the network is the shortest, making it an efficient pathway for information flow. High betweenness signifies that the node lies on the shortest paths between many other node pairs, acting as a bridge or intermediary. These three nodes connect the three components of disease knowledge, lifestyle management, and symptom management. Q18 links disease cognition (Q7, Q16) with specific behaviors (Q11 and Q13), indicating that negative emotions directly impact patients’ self-management adherence. Previous research (29) has revealed the interrelationship between anxiety and depressive symptoms among cardiovascular disease patients, indicating that clinical nurses must integrate psychological assessment and emotional support into routine health education rather than treating them as isolated issues. Q19 represents not only a medical behavior but also serves as a critical node connecting in-hospital and out-of-hospital care, as well as short-term and long-term management. Regular follow-up provides patients with a window to assess the effectiveness of lifestyle adjustments (Q20) and serves as a formal channel for addressing questions about medication efficacy (Q9) and symptom changes (Q16). Q20 is a highly comprehensive question that connects all specific behaviors, including diet (Q11/12), exercise (Q13/14/15), and medication (Q9/10).

### 4.3 Specific implications for nursing practice

The findings of this study offer direct and clear implications for post-PCI nursing practice: 1. Providing a standardized tool: The prompt list developed in this study can serve as a structured conversation checklist for clinical nurses during discharge guidance or outpatient follow-up, ensuring comprehensive and systematic educational content while avoiding omissions of key information. 2. Optimizing educational priorities: Network analysis results suggest nurses should prioritize focusing on core hub issues (such as risk factors and complications) and high-bridging issues (such as emotional management and follow-up examinations), emphasizing detailed explanations and discussions to maximize educational effectiveness. 3. Promoting patient-centered communication: The inherent function of the prompt list is to encourage patient questioning. Nurses should proactively guide patients in using this list, thereby shifting health education from a “nurse-led lecture” model to an interactive “patient inquiry-nurse response” model, better meeting patients’ individualized information needs and enhancing their sense of participation and empowerment. 4. Integrating psychosocial support: Explicitly incorporating “negative emotion management” as a formal component of health education requires nurses to possess basic psychological assessment and support skills or refer patients to psychological resources in a timely manner.

### 4.4 Limitations and future research

This study employed convenience sampling and was conducted in a single hospital. The relatively small sample size may introduce some bias into the research findings. Future studies should adopt multi-center designs and stratified sampling methods. Additionally, as the study relied on cross-sectional data, it can only capture associations between variables without establishing temporal sequence or causal relationships. The reliance on self-reporting or one-time measurements may lead to recall bias or reporting bias, potentially affecting data accuracy. Future research could implement longitudinal designs to investigate changes in patients’ health education needs over time.

## 5 Conclusion

This study, by establishing a list of health education prompts for patients after coronary intervention and conducting network analysis, provides a novel approach to health education for this patient population. Within the entire health education question network, “What are the risk factors for coronary heart disease?” emerged as the most central and patient-concerned health issue. The research indicates the necessity of identifying patients’ health education questions to address disease recurrence and progression caused by knowledge deficits after coronary intervention. Healthcare professionals need to consider each health education question individually and understand their interrelationships when assessing patients and formulating health education strategies. Emphasizing core health education as a vital component of post-intervention care for coronary heart disease is particularly important. The development of this prompt list represents not only an innovation in health education tools but also a significant practice in the transformation of modern nursing models. Its systematic and structured characteristics provide standardized yet flexible educational support for patients after coronary intervention, offering a replicable methodological paradigm for advancing patient-centered care services. Future research may further substantiate the questions through evidence-based approaches and integrate artificial intelligence technologies to enable intelligent delivery of educational content, thereby enhancing the quality of home-based care services.

## Data Availability

The datasets presented in this study can be found in online repositories. The names of the repository/repositories and accession number(s) can be found in the article/supplementary material.
